# Beverage Consumption Habits and Association with Total Water and Energy Intakes in the Spanish Population: Findings of the ANIBES Study

**DOI:** 10.3390/nu8040232

**Published:** 2016-04-20

**Authors:** Mariela Nissensohn, Almudena Sánchez-Villegas, Rosa M. Ortega, Javier Aranceta-Bartrina, Ángel Gil, Marcela González-Gross, Gregorio Varela-Moreiras, Lluis Serra-Majem

**Affiliations:** 1 Research Institute of Biomedical and Health Sciences, University of Las Palmas de Gran Canaria, Las Palmas de Gran Canaria 35016, Spain; marienis67@hotmail.com (M.N.); almudena.sanchez@ulpgc.es (A.S.-V.); 2 CIBER OBN, Biomedical Research Networking Center for Physiopathology of Obesity and Nutrition, Carlos III Health Institute, Madrid 28029, Spain; jaranceta@unav.es (J.A.-B.); agil@ugr.es (A.G.); marcela.gonzalez.gross@upm.es (M.G.-G.); 3 Department of Nutrition, Faculty of Pharmacy, Madrid Complutense University, Madrid 28040, Spain; rortega@ucm.es; 4 Department of Preventive Medicine and Public Health, University of Navarra, Pamplona 31008, Spain; 5 Department of Biochemistry and Molecular Biology II and Institute of Nutrition and Food Sciences, University of Granada, Granada 18100, Spain; 6 ImFINE Research Group, Department of Health and Human Performance, Technical University of Madrid, Madrid 28040, Spain; 7 Department of Pharmaceutical and Health Sciences, Faculty of Pharmacy, CEU San Pablo University, Madrid 28668, Spain; gvarela@ceu.es; 8 Spanish Nutrition Foundation (FEN), Madrid 28010, Spain

**Keywords:** ANIBES, total water intake, energy intake, beverages, Spain

## Abstract

**Background:** Inadequate hydration is a public health issue that imposes a significant economic burden. In Spain, data of total water intake (TWI) are scarce. There is a clear need for a national study that quantifies water and beverage intakes and explores associations between the types of beverages and energy intakes. **Methods:** The Anthropometry, Intake and Energy Balance Study ANIBES is a national survey of diet and nutrition conducted among a representative sample of 2285 healthy participants aged 9–75 years in Spain. Food and beverage intakes were assessed in a food diary over three days. Day and time of beverage consumption were also recorded. **Results:** On average, TWI was 1.7 L (SE 21.2) for men and 1.6 L (SE 18.9) for women. More than 75% of participants had inadequate TWI, according to European Food Safety Authority (EFSA) recommendations. Mean total energy intake (EI) was 1810 kcal/day (SE 11.1), of which 12% was provided by beverages. Water was the most consumed beverage, followed by milk. The contribution of alcoholic drinks to the EI was near 3%. For caloric soft drinks, a relatively low contribution to the EI was obtained, only 2%. Of eight different types of beverages, the variety score was positively correlated with TWI (*r* = 0.39) and EI (*r* = 0.23), suggesting that beverage variety is an indicator of higher consumption of food and drinks. **Conclusions:** The present study demonstrates that well-conducted surveys such as the ANIBES study have the potential to yield rich contextual value data that can emphasize the need to undertake appropriate health and nutrition policies to increase the total water intake at the population level promoting a healthy Mediterranean hydration pattern.

## 1. Introduction

Dehydration occurs when the body loses more water than is taken in. It is often accompanied by disturbances in the body’s mineral salt or electrolyte balance, especially in concentrations of sodium and potassium. Populations at particular risk of hypohydration are children, those engaged in professions where fluid homeostasis is regularly challenged, and older adults [1]. Limited data are available on the prevalence of hypohydration, but there is evidence to suggest that it may be relatively common among older populations [2]. The percentage of the population with inadequate water intake varies from 5% to 35% among European countries [3]. Whereas the burden of disease from inadequate water intake is well known, its consequences in Europe are far from being well understood. Recent research into the risk of disease (from falls and accidents and bowel, metabolic, and kidney diseases), disability (cognitive function, physical performance, and headaches), and death has confirmed the importance of poor hydration with respect to the overall disease burden and quality of life in Europe [4]. Therefore, the significant economic burden dehydration represents makes it an important public health issue. Depending on the degree or magnitude of dehydration in hospitalized patients, costs may be increased by 7%–8.5%. Dehydration represents a potential target for intervention to reduce healthcare expenditures and improve patient quality of life [5].

On the other hand, scientific literature recognizes that the “adequate intake” value of beverages (AI) is a variable event, in which differences are in part due to the inter-individual variation for water needs in response to different health status, metabolism, and environmental factors such as ambient temperature and humidity, as well as individual factors such as age, body size, and level of physical activity. Furthermore, the water needs also depend partially on overall diet and the water contained in food. About 80% of the required daily intake is provided by drinks, including water; the rest is acquired through solid food. Identifying the variety of drinks consumed and establishing the percentage of energy provided by each beverage to the diet allows us to set the pattern of individual or population-level beverage consumption. Understanding the contribution of each fluid type to the total fluid intake will allow us to draw conclusions about the adequacy of drinking habits.

In Spain, the influence of the Mediterranean Diet is widespread. This pattern of consumption also includes the hydration pattern of the population. Traditionally, this pattern included water as a main drink, along with daily but moderate consumption of wine or beer with the principal meal, and the intake of a group of beverages elaborated with fresh vegetables (Gazpacho, Salmorejo, *etc.*). However, in the last decades [6], the adherence to the Mediterranean pattern has been decreasing, especially in children and young people. The actual beverage pattern also included the occasional consumption of soft drinks, which was associated with leisure time. Furthermore, total water intake (TWI) data of Spain are scarce. There are no recent epidemiological studies that focus exclusively on beverage intake. Most available hydration data focuses on alcohol consumption. Apart from the Spanish National Survey on Dietary Intake (ENIDE) [7] in 2011, we are unaware of other research investigating beverage intake among the Spanish population. According to ENIDE data, the average beverage consumption was 1646.5 mL/day, which reflects insufficient fluid intake for that study population.

Given the extent of the problem and its poor recognition, there is the clear need for a national study to update the existing data. The aim of this study was to quantify the total water and beverage intake, and to explore associations between the types of beverage consumed and energy intake. The ANIBES study, a national survey of diet and nutrition conducted in 2013 among a representative random sample of 2285 healthy participants aged 9–75 years, provides us the likely best source of detailed information on the diet of normal individuals in Spain. Characteristics of ANIBES include a food and beverages record list (in grams) of each item consumed per participant and a record of the time of consumption. These study attributes provide a rich resource for exploring patterns of consumption according to age and gender, thereby permitting us to reach conclusions about whether or not drinking a variety of beverages helps to increase fluid intake to a level that meets current guidelines.

## 2. Materials and Methods

The design, protocol, and methodology of the ANIBES study have been described in detail elsewhere [8,9,10].

### 2.1. Sample

The ANIBES study is a cross-sectional study conducted using stratified multistage sampling. To guarantee better coverage and representativeness, the fieldwork was performed at 128 sampling points across Spain. The design of the ANIBES study aims to define a sample size that is representative of all individuals living in Spain, aged 9–75 years, and residing in municipalities of at least 2000 inhabitants. The initial potential sample consisted of 2634 individuals. For all analyses, we eliminated participants with anomalous values of energy intake (EI) (men, <800 or >4000 kcal/day; women, <500 or >3500 kcal/day) [11] to avoid introducing bias to the analysis. The final sample comprised 2007 individuals (1011 men, 50%; 996 women, 50%). In addition, for the youngest age groups (9–12, 13–17, and 18–24 years), an “augment sample” was included to provide at least *n* = 200 per age group (error ± 6.9%). The augment sample is the process of increasing the amount of interviews for a particular subgroup within the population in order to achieve an adequate number of interviews to allow analysis of population subgroups or segments that wouldn't normally yield a sufficient number of interviews in a main random survey, without the expense of increasing the sample size for the whole survey. Therefore, the random sample plus augment sample comprised 2285 participants.

The sample quotas according to the following variables were: age groups (9–12, 13–17, 18–64, and 65–75 years), gender (men/women), and geographical distribution (Northeast, Levant, Southwest, North–Central, Barcelona, Madrid, Balearic, and Canary Islands). Additionally, other factors for sample adjustment were considered: unemployment rate, percentage of foreigners (immigrant population), physical activity level assessed by The International Physical Activity questionnaire (IPAQ) [12], tobacco use and education or economic level. Finally, participants*′* weight, height, and waist circumference were measured and body mass index was also calculated.

The fieldwork for the ANIBES study was conducted from mid-September 2013 to mid-November 2013, and two previous pilot studies were also performed. To equally represent all days of the week, study subjects participated during two weekdays and one weekend day. The final protocol was approved by the Ethical Committee for Clinical Research of the Region of Madrid, Spain [9].

### 2.2. Food and Beverage Record

Study participants were provided with a tablet device (Samsung Galaxy Tab 2 7.0, Samsung Electronics, Suwon, South Korea) and trained in recording information by taking photos of all food and drinks consumed during the 3 days of the study, both at home and outside the home. Photos were to be taken before beginning to eat and drink, and again after finishing, so as to record the actual intake. Additionally, a brief description of meals, recipes, brands, and other information was recorded using the tablet. Participants who declared or demonstrated that they were unable to use the tablet device were offered other options, such as using a digital camera and paper record and/or telephone interviews. A total 79% of the sample used a tablet, 12% a digital camera, and 9% opted for a telephone interview. As no differences in the percentage of misreporting were found according to the type of device used to assess dietary intake, we used the measurements of the three assessment methods in the analysis. In addition to details of what and how much was eaten, for each eating/drinking event, participants recorded where they were, who they were eating with, and whether they were watching television and/or sitting at a table. After each survey day, participants recorded if their intake was representative for that day (or the reason why if it was not), and details of any dietary supplements taken. The survey also contained a series of questions about participants*′* customary eating habits (e.g., the type of milk usually consumed) to facilitate further coding. Food records were returned from the field in real time, to be coded by trained coders who were supervised by dieticians. An *ad hoc* central server software/database was developed for this purpose, to work in parallel with the codification and verification processes. Food, beverage, and energy and nutrient intakes were calculated from food consumption records using VD-FEN 2.1 software, a Dietary Evaluation Program from the Spanish Nutrition Foundation (FEN), Spain, which was newly developed for the ANIBES study by the FEN and is based mainly on Spanish food composition tables [13], with several expansions and updates. Data obtained from food manufacturers and nutritional information provided on food labels were also included. A food photographic atlas was used to assist in assigning gram weights to portion sizes. The VD-FEN 2.1 software was developed to receive information from field tablets every 2 s, and the database was updated every 30 min. Energy distribution objectives for the Spanish population were used to analyze the overall quality of the diet [9,14].

### 2.3. Data Preparation and Analysis

The present analysis focused on the TWI of all food and drink, determined from food composition tables with several adaptations and updates [13]. Metabolic water (water derived from oxidation of substrates) was not included so as to focus on comparisons with dietary water requirements.

Beverages were combined into eight categories for further analysis: (1) hot beverages, including hot tea and coffee (iced teas in cans or bottles were considered caloric soft drinks); (2) milk (all types of milk without separation by fat percentage); (3) fruit and vegetable juices (including nectars, juice–milk blends, 100% fruit juices, and some typical Spanish beverages: horchata, gazpacho, salmorejo, and white garlic); (4) caloric soft drinks (including colas, tonic water, sodas, ginger ale, fruit flavored drinks, iced teas in cans or bottles, sports drinks such as isotonic drinks with mineral salts, and caffeinated energy drinks); (5) diet soft drinks (including the same beverages as in the caloric soft drinks group but with artificial sweetener); (6) alcoholic drinks, including two groups: (a) low-alcohol grade (mostly beer, wine, and cider); and (b) high-alcohol grade (including brandies, liqueurs, tequila, vodka, whisky, *etc.*); (7) water (including tap water and bottled water); and (8) other beverages (including soy-based beverages, non-alcoholic beer and wine, and others).

Additionally, a variety score was created as the sum of the different beverages used in our classification with a minimum value of 0 and a maximum value of 8.

To investigate daily trends, beverage consumption events were aggregated into six time periods, approximately corresponding to breakfast (up to 10:00 a.m.), mid-morning (10:00 a.m.–1:00 p.m.), lunch (1:00 p.m.–4:00 p.m.), mid-afternoon (4:00 p.m.–7:00 p.m.), dinner (7:00 p.m.–10:00 p.m.), and other times.

TWI was compared with the European Food Safety Authority (EFSA) Dietary Reference Values (DRV) for the Adequate Intake (AI) of water for men and women from 14 years of age onward (2.5 L and 2.0 L, respectively), and for boys and girls from 9 to 13 years of age (2.1 L and 1.9 L, respectively) [15]. Furthermore, Nordic and German-speaking countries take the approach that water intake is considered inadequate when it is less than 1 g per kilocalorie of energy requirement [15]. Therefore, we used three different approaches to define water intake adequacy to provide a more comprehensive estimate of the proportion of participants who consume low amounts of water [16]. First, a classification based on the AI value, defined by the EFSA as criterion 1. The second (criterion 2), a ratio between TWI (water from food and beverages in grams) and EI in kcal higher than 1; and the combination of both as final criterion.

### 2.4. Statistical Analyses

Owing to reported differences in water consumption and recommended intakes between male and female individuals, all analyses were carried out separately by gender. Crude differences in TWI and beverage consumption between groups were assessed through an analysis of variance test or *t*-tests with Bonferroni correction for multiple comparisons. Chi-squared tests were used for categorical variables. All analyses were two-tailed, with statistical significance set at *p* < 0.05.

Partial Correlations between water intake, energy intake, and beverage consumption adjusted for age, gender, body weight, and physical activity were calculated by the use of a variety score.

Multivariable linear regression models included adjustment for gender, age, weight, and physical activity level. Multiple linear regression models were fitted to assess the effect of varying the type of beverages consumed (caloric *vs.* non-caloric) on EI while controlling for the effect of confounders (gender, age, weight, and physical activity). The effect of replacing 100 g of caloric beverages with 100 g of non-caloric beverages was estimated by including caloric beverages (as a percentage of total beverage weight) as the main independent variable, with total beverage weight (g) held constant. This necessarily implies an equal and opposite change in other beverages. A further model included energy from food, thus disallowing compensation (reduction in calories from food). Finally, we used within-person daily consumption data to explore the effect of changes in daily beverage consumption, with each person acting as their own control. The independent variables represent the standard deviation of the mean of the three different measurements (3-day records) for each type of beverage collected for each participant. The outcome (change in EI) was derived using the same methodology (deviation from participants*′* 3-day mean in energy intake) [16].

## 3. Results

The ANIBES sample was of 2285 healthy subjects aged 9 to 75 years, of which 50% of the population was men and the other 50% were women. The study sample reflects the distribution of male and female individuals in the general population of Spain [17] (Statistics National Institute (INE) 2011). A more detailed description of the ANIBES study population is given in Table 1 [13]. There was no statistically significant difference in the education or economic level between men and women. However, men smoked more but engaged in more sport than women. Regarding the measures of overweight and obesity, the data of ANIBES study were representative of the Spanish adult population, according to the Spanish National Health Survey 2011–2012 [18]: 37% and 32% of men and women, respectively, were overweight, and 21% and 19%, respectively, were obese.

**Figure 1:**
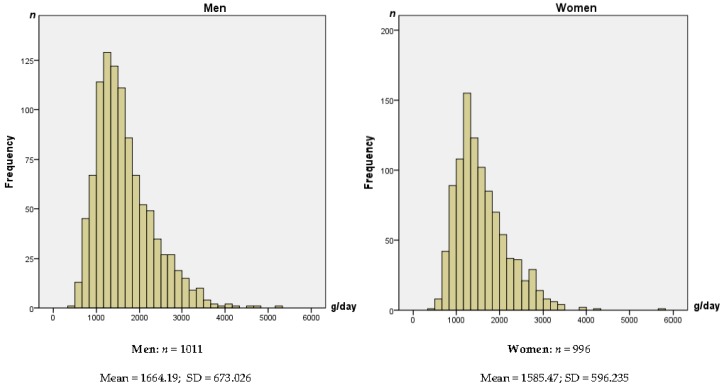
Frequency distribution of total water intake (g/day) over three days by gender.

Figure 1 shows the frequency distribution of TWI (g/day) over a three-day recording period, organized by gender. On average, the TWI was 1.7 L (SE 21.2) for men and 1.6 L (SE 18.9) for women, far less than the EFSA AI recommendations for adults (2.5 L and 2.0 L, respectively).

Percentages of total weight consumed (g/day), daily EI (kcal/day), and water intake (g/day) are presented in Table 2. Beverages were separated by category, with most consumed in similar amounts by participants of both genders. However, men consumed exactly two times more alcoholic beverages than women. The mean total EI was 1809 kcal/day (SE 11.1), and the relative contribution to total EI from beverages was 12% (13% for men, 12% for women). Furthermore, 68% of the TWI came from beverages and 32% from food. Those amounts coincided with EFSA recommendations (70%–80% provided by beverages of all types and the remaining 20%–30% from food).

**Figure 2:**
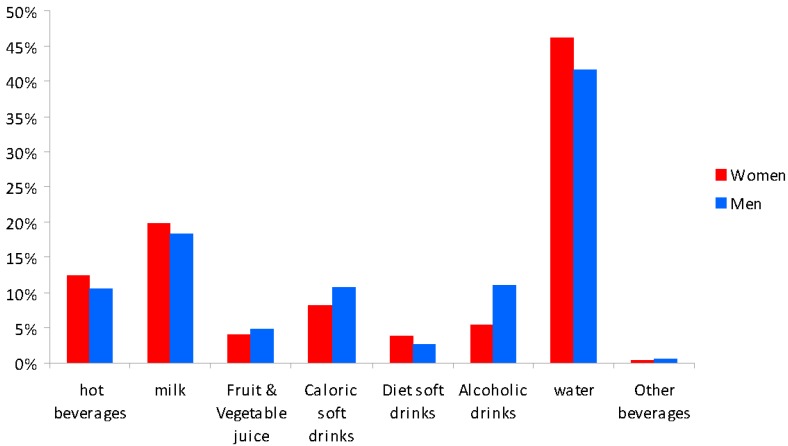
Percentage of beverages consumed over a three-day period by gender.

On average, the percentage of total beverage consumption from water over the three-day study period was 46% for women and 41% for men (Figure 2). Water was the most frequently consumed beverage, followed by milk, for both genders. Among men, the decreasing order of consumption was alcoholic drinks, caloric soft drinks, and hot beverages, with similar percentages (11%, 11%, and 10%, respectively). For women, the decreasing order was hot beverages (12%), caloric soft drinks (8%), and alcohol (5%). Fruit and vegetable juices and diet soft drinks were consumed in lower amounts by both genders.

In general (Table 3), the contribution of water intake from food increased with age, from 434 g/day (SE 13.9) among younger participants (13–17 years) to 584 g/day (SE 24.8) among older adults (65–75 years). This finding is likely owing to lower consumption of fruits and vegetables, which are rich in water, for the youngest participants. The water contribution from beverages declined from 1202 g/day (SE 21.6) among adults (18–64 years) to 1002 g/day (SE 44.0) among older adults.

For adolescents, the mean consumption of caloric soft drinks was 167 g/day (SE 19.1) for men, and 139 g/day (SE 15.9) for women (equivalent to about three cans per week for each). However, consumption was lower among adults, with 114 g/day (SE 6.3) for men and 82.1 g/day (SE 4.6) for women. Consumption of alcoholic drinks for adult men and women averaged 160 g/day (SE 9.0) and 71 g/day (SE 4.8), respectively. The intake of alcoholic drinks among the oldest participants (65–75 years) averaged 143 g/day (SE 19.0) for men and 53 g/day (SE 9.8) for women.

**Figure 3:**
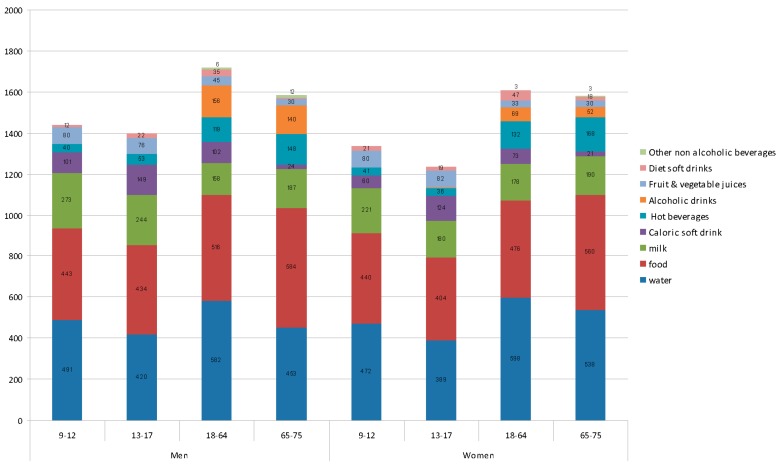
Daily Water intakes (g/day) from beverage/food categories by age and gender among ANBIES population.

The principal sources of total dietary water, by gender and age group, are shown in Figure 3. For both men and women, the main source was water, followed by milk. Children and adolescents consumed higher amounts of water from milk and juices than adults, for both genders; however, adolescents consumed less water from hot beverages and diet soft drinks than adults. Adults and older adults had a lower intake of caloric soft drinks and juices but consumed greater amounts of hot beverages and alcohol, for both men and women.

TWI was highly correlated with beverage weight and water from beverages (*r* = 0.95), and was more weakly correlated with food intake (*r* = 0.54, Table 4). Caloric soft drinks and alcoholic drinks had a moderate correlation with total energy from beverages (*r* = 0.47 and 0.49, respectively), whereas coefficients were lowest for hot beverages and diet soft drinks.

Regarding beverage variety of eight types of beverages in our classification, the variety score was positively correlated with TWI (*r* = 0.39, *p* < 0.001) and with EI (*r* = 0.23, *p* < 0.001), suggesting that beverage variety is an indicator of a higher consumption of food and drinks.

**Figure 4:**
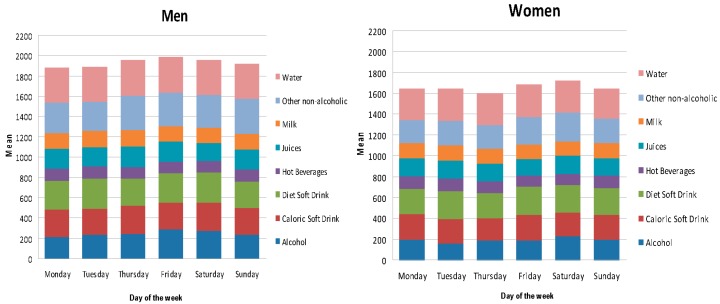
Amount and types of beverages consumed according to day of the week (mean g/day), separated by gender.

Figure 4 represents the influence of day of the week on beverage consumption. The total amount of beverage intake (g) was slightly higher on Fridays among men and on Saturdays among women than on other days of the week. This appears to be attributable to a higher consumption of alcoholic drinks on the weekends for both groups. Consumption of water, milk, fruit and vegetable juices, hot beverages, and diet soft drinks did not vary greatly by day of the week. With respect to beverage consumption according to the time of day, over a 24 h period, in general, no significant differences were found by age or time of day in either men or women (Table 5). One exception was seen in differences in the amount of beverages consumed by males during lunch when comparing children and adolescents to adults, the consumption is higher in 18–64 year-old subjects.

Appendix Table A1 shows a multiple regression model including four covariates (gender, age, weight, and physical activity) as well as total beverage intake, which is mathematically equivalent to substituting caloric beverages (sum of hot beverages, milk, fruit and vegetable juices, caloric soft drinks, and alcoholic drinks) with an equal weight of non-caloric beverages (diet soft drinks and water). The predicted effect of replacing 100 g of caloric beverages with 100 g of non-caloric drinks was associated with a reduction in EI of 50 kcal (Model 1). When food EI was constrained as a constant (*i.e.*, disallowing compensation), the net impact of 100 g of caloric beverages was estimated at 40 kcal (Model 2). Further adjustment for level of education, economic level, employment, *etc.* did not change the reported results.

The second regression model analysis (Appendix Table A2) used a within-person change model to address whether a change in a participants*′* beverage consumption habits on any day could be associated with a change in their total EI (compared with their three-day mean). Modeling each beverage separately, with total beverage weight held constant, each of the above two non-caloric beverages (diet soft drink and water) was negatively associated with energy (as were hot beverages), whereas the five caloric beverages mentioned above were positively associated with energy (Model 3). When combining caloric beverages, the final models (Models 4 and 5) gave an estimated effect of 43 kcal per 100 g of caloric drinks substituted, or 34 kcal if EI from food was held constant.

Participants who fulfilled the EFSA AI recommendations of TWI for men and women (2.5 L and 2.0 L, respectively) were classified as Criterion 1. Participants with ratios of water/energy intake >1.0 were included as Criterion 2 (considering a value of 1 g of water per 1 kcal of energy intake). Finally, participants who met both definitions (Criteria 1 and 2) were classified as Criterion 3. For children 9–13 years old, the EFSA AI is 2.1 L for boys and 1.9 L for girls. Following this analysis, Table 6 shows that for both genders, more than 75% of women, about 80% of men, and nearly 90% of children did not meet the AI recommendation for water consumption.

## 4. Discussion

This study provides analyses of total water intakes from all sources among a nationally representative sample of the Spanish population aged 9–75 years, included in the 2013 ANIBES study database. To our knowledge, the present analyses represent one of the few explorations of the consumption of water and beverages in Spain, as well as the association with energy intake, consumption according to time of day and day of the week, the association between beverage variety and increased fluid intake, and compliance with current AI recommendations, by gender and age.

The main findings of this study indicate that for the entire sample, TWI was 1625 g/day (SE 14.2). In general, and as expected, male individuals had statistically higher intakes than female individuals for both food and beverages. However, neither men nor women consumed sufficient amounts of water (1664 g/day for men, 1585 g/day for women), according to EFSA AI reference values [15]. Men consumed approximately 33% less than the AI and women nearly 21% less.

Most of the data analysis in this work was based on an earlier survey by Gibson and Shirreffs [16], who analyzed the weighed dietary records from the National Diet and Nutrition Survey (2000/2001) of 1724 British adults. Contrary to our findings, mean TWI in that population was nearly identical to the EFSA reference AI for both genders.

The comparison is difficult to do when we want to compare American and Spanish populations. The NHANES study, developed in the US between 2005 and 2010, used the proposal by the Institute of Medicine of the United States of America (IOM) as reference value, set as 3.7 L/day for men and 2.7 L/day for women [19]. This leads us to consider the need to investigate why the recommendations of the EFSA and the IOM are so different if both recommendations include the water from both food and beverage sources. The clarification of this issue deserves further study.

Mean daily EI was 1809 kcal/day (SE 11.1), of which 12% was provided by the beverages, close to the 10% proposed by some international authorities (EFSA, WHO) [15,20] who recommended that no more than 10% of the daily calorie intake should come from beverages. In the NHANES study [21], the proportion of energy from beverages was 21%, while it was 16% in the British study [16]. Regarding this issue, in recent years, the impact of caloric soft drink consumption on obesity and metabolic disorders has come under intense scrutiny and debate worldwide [22,23,24,25], and large differences between countries have been observed. The present study showed that for the entire Spanish population, caloric soft drinks contributed only 2% of the total EI, or 41.4 kcal/day (SE 1.5) out of a total EI of 1809 kcal/day (SE 11.1). Lower consumption was seen in older adults, at 26.5 g/day (SE 6.6). Higher consumption was found among adolescents, at 167 g/day (SE 19.1), followed by adults and children, with similar mean consumptions of 114.2 g/day (SE 6.3) and 113 g/day (SE 14.8), respectively). This finding is perhaps one of the most interesting findings of our study. The relatively low contribution of caloric soft drinks to the EI could be attributable to the Mediterranean pattern of consumption that this society keeps. By contrast, the NHANES study of the United States [21] has the highest contribution to EI from sweetened beverages. For adults, soda accounted for near 6% of energy intake.

On the other hand, beverage consumption is uneven throughout the day; in this study, it tended to be concentrated at lunchtime and was slightly higher on Fridays in men and on Saturdays in women. Although we did not find large variation, these findings reflect certain cultural trends of consumption that appear to be attributable to a higher intake of alcoholic beverages on weekends among both genders. The contribution of alcoholic drinks to the diet in the ANIBES study (135.7 g/day, SE 7.5) was slightly higher than that in the ENIDE dietary survey of Spanish adults in 2011 (117 g/day) [7]. It is important to note that beverages with lower alcohol content (beer, wine, and cider) represented over 90% of the energy contribution in adult and older populations. Furthermore, although these data are of the highest quality obtainable, alcohol intake is one of the dietary components for which underreporting may occur, especially among women and participants with higher education and socioeconomic levels [26,27]. In fact, the reported amount of alcohol consumed by men was twice that reported by women in this study. Anyway, this relatively high consumption of alcohol shown could also be attributable to the Mediterranean dietary pattern that certainly remains rooted in Spanish society. The Spanish Society of Community Nutrition (SENC) recommends the maximum consumption of 1 to 1.5 servings/day of alcoholic beverages in women and 2 to 2.5 servings/day for adult males in the context of a Mediterranean balanced diet. Consumption of alcoholic beverages is associated with a more or less healthy dietary pattern. The types of alcoholic beverages that are closer to the Mediterranean environment are [28] fermented drinks, wine, beer, and cider consumed during the principal meals.

The strengths of this study include the careful design, protocol, and methodology used in the ANIBES study. The present analysis can be used to inform approaches to improving the overall quality of diet and hydration status of the Spanish population.

Two important limitations of this study must be noted. This study is a cross-sectional design, which provides evidence for association but not causal relationships. The second limitation is that, for logistical reasons, there was no inclusion of a hydration biomarker, which would allow assessment of dietary beverage intake and hydration status without the bias of self-reported dietary intake and also intra-individual variability. This biomarker should be included in further studies.

## 5. Conclusions

The present study shows clearly that neither men nor women consumed adequate TWI when compared with EFSA reference values. The ANIBES study demonstrated that well-conducted national surveys have the potential to yield rich contextual value data that can emphasize the need to undertake appropriate health and nutrition policies to increase the total water intake at the population level, promoting a healthy Mediterranean hydration pattern.

## Figures and Tables

**Table 1:** Statistical description of the sample ANIBES.

				Total	%	Male	%	Female	%
			Total	2007	100	1011	50.4	996	49.6
Age Group		9–12	Count	100	5	62	6.1	38	3.8
		13–17	Count	123	6.1	84	8.3	39	3.9
		18–39	Count	777	38.7	387	38.3	390	39.2
		40–64	Count	810	40.4	385	38.1	425	42.7
		65–75	Count	197	9.8	93	9.2	104	10.4
Unemployed			Count	270	13.5	184	18.2	86	8.6
Foreigners (immigrant population)	Spanish		Count	1933	96.3	975	96.4	958	96.2
	Foreign		Count	74	3.7	36	3.6	38	3.8
Level of physical activity	Inactive		Count	884	44.0	389	38.5	495	49.7
	Active		Count	1123	56.0	622	61.5	501	50.3
Level of education	Primary or less		Count	743	37.0	378	37.4	365	36.6
	Secondary		Count	858	42.8	434	42.9	424	42.6
	Tertiary or University		Count	406	20.2	199	19.7	207	20.8
Economical level	1000 € or less		Count	397	19.8	191	18.9	206	20.7
	From 1000 to 2000 €		Count	795	39.6	393	38.9	402	40.4
	Over 2000 €		Count	320	15.9	163	16.1	157	15.8
	No income		Count	7	0.3	4	0.4	3	0.3
	No answer		Count	488	24.3	260	25.7	228	22.9
Geographical distribution	Northwest		Count	152	7.6	77	7.6	75	7.5
	North Central		Count	161	8.0	79	7.8	82	8.2
	Northeast + Barcelona AAMM		Count	368	18.3	177	17.5	191	19.2
	Center + Madrid AAMM		Count	455	22.7	240	23.7	215	21.6
	Levante		Count	335	16.7	176	17.4	159	16.0
	South		Count	443	22.1	218	21.6	225	22.6
	Canarias		Count	93	4.6	44	4.4	49	4.9
Tabaco	Yes		Count	602	30.0	338	33.4	264	26.5
	No		Count	1182	58.9	527	52.1	655	65.8
Weight (kg)			Mean (SE)	72.30 (0.39)		78.40 (0.56)		66.10 (0.46)	
Height (cm)			Mean (SE)	166.20 (0.23)		172.10 (0.31)		160.20 (0.22)	
Waist Circumference			Mean (SE)	87.70 (0.34)		91.90 (0.48)		83.40 (0.45)	
BMI class (kg/m^2^)	Underweight		Count	27	1.34	5	0.5	22	2.2
	Normal weight		Count	880	43.84	417	41.2	463	46.5
	Overweight		Count	694	34.57	375	37.1	319	32.0
	Obese		Count	406	20.22	214	21.2	192	19.3

**Table 2:** Contribution of food and beverages to total water and energy intake.

		Total Weight Consumed (g/Day) GRAMS			Contribution to Energy Intake (kcal/Day) KCAL			Contribution to Water Intake (g/Day) WATER		
		Total	Men	Women	Total	Men	Women	Total	Men	Women
	Count	2007	1011	996	2007	1011	996	2007	1011	996
All food and drink	Mean (SE)	2071.55	2136.35	2005.77	1809.01	1955.68	1660.15	1625.12	1664.19	1585.47
		15.87	23.81	20.74	11.15	16.43	13.52	14.22	21.17	18.89
Food only	Mean (SE)	45.0%	45.5%	44.6%	87.8%	87.2%	88.4%	32.2%	32.4%	32.1%
		0.3%	0.4%	0.4%	0.1%	0.2%	0.2%	0.3%	0.3%	0.4%
Beverages only	Mean (SE)	55.0%	54.5%	55.4%	12.2%	12.8%	11.6%	67.8%	67.6%	67.9%
		0.3%	0.4%	0.4%	0.1%	0.2%	0.2%	0.3%	0.3%	0.4%
Hot beverages	Mean (SE)	6.1%	5.5%	6.6%	0.4%	0.4%	0.5%	7.7%	7.0%	8.3%
		0.1%	0.2%	0.2%	0.0%	0.0%	0.0%	0.2%	0.2%	0.3%
Milk	Mean (SE)	10.0%	9.5%	10.6%	5.6%	5.1%	6.0%	11.8%	11.3%	12.3%
		0.2%	0.2%	0.2%	0.1%	0.1%	0.1%	0.2%	0.3%	0.3%
Fruit & Vegetable Juices	Mean (SE)	2.4%	2.6%	2.2%	1.3%	1.4%	1.2%	2.8%	3.0%	2.5%
		0.1%	0.2%	0.1%	0.1%	0.1%	0.1%	0.1%	0.2%	0.2%
Caloric soft drink	Mean (SE)	5.1%	5.8%	4.4%	2.2%	2.4%	2.0%	6.1%	6.9%	5.2%
		0.2%	0.3%	0.2%	0.1%	0.1%	0.1%	0.2%	0.4%	0.3%
Diet soft drink	Mean (SE)	1.8%	1.4%	2.1%	0.0%	0.0%	0.0%	2.3%	1.8%	2.7%
		0.1%	0.1%	0.2%	0.0%	0.0%	0.0%	0.1%	0.2%	0.2%
Alcohol	Mean (SE)	4.6%	6.2%	3.0%	2.7%	3.5%	1.9%	5.7%	7.6%	3.7%
		0.2%	0.3%	0.2%	0.1%	0.2%	0.1%	0.2%	0.4%	0.2%
Water	Mean (SE)	24.8%	23.3%	26.3%	-	-	-	31.2%	29.5%	32.9%
		0.4%	0.5%	0.5%	-	-	-	0.4%	0.6%	0.6%
Other non-alcoholic beverages	Mean (SE)	0.2%	0.3%	0.2%	0.0%	0.0%	0.0%	0.3%	0.3%	0.2%
		0.0%	0.1%	0.0%	0.0%	0.0%	0.0%	0.0%	0.1%	0.0%

**Table 3:** Total water intake and beverage consumption (g/day) by sex and age group (*n* = 2281).

	Men						Women					
	Age Group					*p* ^1^	Age Group					*p* ^1^
	Base	9–12	13–17	18–64	65–75		Base	9–12	13–17	18–64	65–75	
	(A)	(B)	(C)	(D)	(E)		(F)	(G)	(H)	(I)	(J)	
Base	1156	125	136	796	99		1125	87	74	857	107	
Total Water intake from food & beverage Mean (SE)	1638.63 (19.40)	1440.20 ^(a)^ (45.47)	1398.04 ^(b)^ (43.29)	1717.49 (24.69)	1585.57 (57.75)	<0.001	1559.94 (17.53)	1334.55 ^(h)^ (46.58)	1235.51 ^(f,g)^ (40.07)	1608.35 (20.97)	1579.77 (48.94)	<0.001
Water from Food Mean (SE)	503.90 (6.07)	443.62 ^(a,c)^ (13.72)	433.60 ^(b,d)^ (13.96)	515.60 ^(e)^ (7.41)	583.72 (24.81)	<0.001	476.31 (5.67)	440.18 (19.42)	403.67 ^(g,i)^ (17.83)	475.75 ^(j)^ (6.45)	560.47 (19.59)	<0.001
Water from beverages only Mean (SE)	1134.73 (16.74)	997.58 ^(a)^ (39.33)	964.44 ^(b)^ (36.31)	1201.89 ^(e)^ (21.61)	1001.85 (44.03)	<0.001	1083.62 (15.55)	894.37 ^(h)^ (40.30)	831.84 ^(f)^ (33.16)	1132.60 (18.72)	1019.31 (42.33)	<0.001
Total beverages Consumption Mean (g/day) (SE)	1181.00 (16.94)	1058.60 ^(a)^ (40.39)	1026.43 ^(b)^ (37.54)	1244.72 ^(e)^ (21.86)	1035.52 (44.79)	<0.001	1121.62 (15.65)	943.92 ^(i)^ (41.27)	881.93 ^(f)^ (33.59)	1169.24 (18.85)	1050.49 (42.78)	<0.001
OF WHICH (g/day)												
Hot beverages Mean (g/day) (SE)	107.14 (3.60)	41.20 ^(a,c)^ (4.26)	54.43 ^(b,d)^ (7.37)	120.95 (4.55)	151.82 (12.91)	<0.001	123.93 (3.96)	41.85 ^(h,i)^ (6.12)	37.15 ^(f,g)^ (6.81)	134.00 (4.33)	170.00 (19.02)	<0.001
Milk Mean (g/day) (SE)	204.48 (4.52)	309.73 ^(a,c)^ (12.00)	274.67 ^(b,d)^ (16.00)	175.69 (4.94)	206.64 (15.24)	<0.001	202.55 (3.85)	249.77 ^(h)^ (14.51)	201.86 (15.43)	196.82 (4.28)	210.45 (14.12)	0.003
Fruit & Vegetable Juices Mean (g/day) (SE)	58.22 (3.15)	91.56 ^(a,c)^ (11.83)	87.67 ^(b,d)^ (11.31)	50.94 (3.51)	34.25 (7.74)	<0.001	45.05 (2.42)	91.76 ^(h,i)^ (12.08)	93.35 ^(f,g)^ (17.17)	37.46 (2.27)	34.42 (6.66)	<0.001
Caloric soft drink Mean (g/day) (SE)	112.76 (5.25)	113.02 ^(c)^ (14.76)	166.97 ^(b,d)^ (19.06)	114.19 ^(e)^ (6.32)	26.43 (6.57)	<0.001	79.17 (3.86)	67.32 (11.10)	138.94 ^(g)^ (15.91)	82.15 ^(j)^ (4.59)	23.66 (6.18)	<0.001
Diet soft drink Mean (g/day) (SE)	28.81 (2.70)	12.09 ^(a,c)^ (3.17)	21.65 ^(b)^ (6.41)	35.25 ^(d)^ (3.68)	7.98 (3.13)	0.003	40.26 (3.56)	20.90 (6.48)	18.56 (9.28)	46.88 (4.48)	17.91 (6.16)	0.011
Alcohol Mean (g/day) (SE)	122.36 (6.72)	- -	1.14 ^(b,d)^ (1.14)	159.66 (9.05)	143.45 (19.00)	<0.001	59.03 (3.88)	- -	2.93 (2.34)	70.64 (4.85)	52.82 (9.78)	<0.001
Water Mean (g/day) (SE)	542.12 (14.81)	491.00 (35.99)	419.88 ^(b)^ (33.19)	582.15 (19.15)	452.69 (40.51)	<0.001	568.75 (14.17)	472.31 (39.27)	389.14 ^(f)^ (34.77)	597.92 (17.03)	537.72 (40.88)	<0.001
Other non alcoholic beverages (g/day) (SE)	5.11 (1.11)	-	-	5.90 (1.49)	12.26 (5.05)	*0.034*	2.90 (0.55)	- -	- -	3.347 (0.67)	3.52 (2.14)	0.195

*p* ^1^ value obtained through ANOVA test; ^(a)^ BD; ^(b)^ CD; ^(c)^ BE; ^(d)^ CE; ^(e)^ DE; ^(f)^ HI; ^(g)^ HJ; ^(h)^ GI; ^(i)^ GJ; ^(j)^ IJ = Significant.

**Table 4:** Partial correlations between water intake, energy intake and beverage consumption. (3 days mean data adjusted for age, gender, body weight and physical activity).

	Total Water (from Food & Beverages) (g/Day)	Total Water from Beverages (g/Day)	Total Water from Food (g/Day)	Total Food Weight (g/Day)	Total Beverages Weight (g/Day)	Total Energy (kcal)	Total Energy from Food (kcal)	Total Energy (kcal) from Beverages
Total Water (g/day) (from food and beverages)	1	0.952 **	0.524 **	0.542 **	0.950 **	0.411 **	0.376 **	0.258 **
Total Water (g/day) from beverages	0.952 **	1	0.239 **	0.271 **	0.999 **	0.315 **	0.256 **	0.301 **
Total Water (g/day) from food	0.524 **	0.239 **	1	0.966 **	0.233 **	0.428 **	0.480 **	−0.017
Total Food weight (g/day)	0.542 **	0.271 **	0.966 **	1	0.268 **	0.594 **	0.647 **	0.042
Total Beverages weight (g/day)	0.950 **	0.999 **	0.233 **	0.268 **	1	0.331 **	0.264 **	0.335 **
Total energy (kcal)	0.411 **	0.315 **	0.428 **	0.594 **	0.331 **	1	0.962 **	0.477 **
Total Energy (kcal) from food	0.376 **	0.256 **	0.480 **	0.647 **	0.264 **	0.962 **	1	0.219 **
Total Energy (kcal) from beverages	0.258 **	0.301 **	−0.017	0.042	0.335 **	0.477 **	0.219 **	1
(1) Hot beverages (g/day)	0.241 **	0.220 **	0.153 **	0.145 **	0.214 **	0.001	0.008	−0.022
(2) Milk (g/day)	0.160 **	0.170 **	0.036	0.069 **	0.189 **	0.199 **	0.111 **	0.356 **
(3) Fruit & vegetable juice (g/day)	0.086 **	0.093 **	0.015	0.028	0.113 **	0.154 **	0.074 **	0.315 **
(4) Caloric soft drink (g/day)	−0.028	0.009	−0.115 **	−0.072 **	0.038	0.273 **	0.157 **	0.471 **
(5) Diet soft drink (g/day)	0.068 **	0.096 **	−0.052^*^	−0.040	0.093 **	0.000	0.023	−0.074 **
(6) Alcohol (g/day)	0.271 **	0.304 **	0.014	0.031	0.304 **	0.193 **	0.061 **	0.494 **
(7) Water (g/day)	0.830 **	0.857 **	0.249 **	0.256 **	0.843 **	0.127 **	0.171 **	−0.097 **
(8) Other non alcoholic beverages (g/day)	0.064 **	0.056 *	0.049 *	0.048 *	0.054 *	0.015	0.021	−0.015
Variety of beverages consumed in day (out of 8)	0.392 **	0.401 **	0.130 **	0.156 **	0.408 **	0.229 **	0.143 **	0.360 **

** Correlation is significant at the 0.01 level (bilateral); * Correlation is significant at the 0.05 level (bilateral).

**Table 5:** Beverage consumption according to time of day (hour interval), by age and gender.

Amount of Beverages (g/Day) Consumed between	Men						Women					
	Age Group						Age Group					
	9–12 (A)	13–17 (B)	18–64 (C)	65–75 (D)	Total	*p* ^1^	9–12 (A)	13–17 (B)	18–64 (C)	65–75 (D)	Total	*p* ^1^
Breakfast <10:00 Mean (SE)	231.90 (6.84)	233.10 (9.50)	223.50 (4.45)	238.70 (12.47)	226.90 (3.50)	*0.532*	217.70 (10.54)	194.10 ^(d)^ (10.24)	233.50 (4.47)	256.40 (13.47)	232.00 (3.81)	*0.01*
Mid-morning 10:00 to 13:00 Mean (SE)	160.50 (17.28)	153.60 (17.17)	173.90 (6.98)	140.50 (13.23)	167.50 (5.66)	*0.301*	120.40 (11.25)	126.40 (13.68)	149.10 (6.19)	149.30 (14.86)	146.10 (5.20)	*0.436*
Lunch 13:00 to 16:00 Mean (SE)	283.90 ^(a)^ (12.72)	295.70 ^(b)^ (14.02)	344.80 (6.32)	303.40 (16.00)	328.80 (5.09)	*<0.001*	248.30 (14.33)	268.50 (12.20)	285.10 (5.15)	274.50 (12.42)	280.10 (4.32)	*0.116*
Snack 16:00 to 19:00 Mean (SE)	166.30 (12.66)	135.00 (8.71)	167.00 (6.79)	134.40 (14.28)	160.60 (5.10)	*0.1*	147.10 (12.22)	124.40 (10.73)	138.80 (5.15)	172.10 (14.23)	142.00 (4.32)	*0.096*
Dinner 19:00 to 22:00 Mean (SE)	248.30 ^(a)^ (9.86)	278.00 (14.16)	301.70 (6.02)	259.10 (13.85)	289.50 (4.77)	*<0.001*	229.80 (13.37)	252.40 (20.04)	248.20 (4.72)	237.80 (14.76)	246.10 (4.20)	*0.593*
Other moments Mean (SE)	237.80 ^(a)^ (34.62)	309.80 (50.88)	444.20 ^(c)^ (25.25)	218.70 (37.67)	392.40 (19.78)	*<0.001*	180.80^(a)^ (27.13)	140.50 ^(b)^ (11.01)	397.90 (17.71)	311.50 (47.13)	364.10 (15.19)	*<0.001*

*p* ^1^ value obtained through ANOVA test. (a) AC; (b) BC; (c) CD; (d) BD = significant.

**Table 6:** Combined classification for the Total water intake (TWI) following established criteria.

**EFSA 2.5 L Men, 2.0 L Women (14 a 75 Years) *n* = 2023**	**Men**	**Women**
CRITERION 1: *n* (%)	241 (12)	431 (21)
CRITERION 2: *n* (%)	538 (27)	805 (40)
CRITERION 3 (1 and 2): *n* (%)	202 (10)	370 (18)
**EFSA 2.1 L Boys, 1.9 L Girls (9 a 13 Years) *n* = 258**	**Boys**	**Girls**
CRITERION 1: *n* (%)	28 (11)	28 (11)
CRITERION 2: *n* (%)	25 (10)	28 (11)
CRITERION 3 (1 and 2): *n* (%)	13 (5)	18 (7)

EFSA: European Food Safety Authority. (1) Criterion 1: TWI >2.5 L men, >2 L women (aged 14 to 75 years) >2.1 L boys, >1.9 L girls (ages 9 to 13 years); (2) Criterion 2: Ratio of total water/total energy intakes >1; (3) Criterion 3: Both criteria.

## Appendix

**Table A1:** Regression model estimating change in energy intake associated with adding caloric beverages in place of non-caloric beverages.

**Model 1**					
		**B**	***p* Value**	**95% Confidence Interval for B**	
				**Lower**	**Upper**
(Constant)		1597.476	<0.001	1507.809	1687.144
Gender	men	253.295	<0.001	211.937	294.653
Age (years)		−4.715	<0.001	−5.931	−3.499
Weight (kg)		−2.722	<0.001	−3.956	−1.488
Physical activity	active	−34.556	0.092	−74.704	5.592
Total beverages (100 g/day)		18.191	<0.001	14.541	21.841
5caloricbeverages (100 g) *		49.817	<0.001	42.757	56.877
**Model 2: Keeping Food Constant**					
		**B**	***p* Value**	**95% Confidence Interval for B**	
				**Lower**	**Upper**
(Constant)		46.798	<0.001	27.063	66.533
Gender	men	22.655	<0.001	15.224	30.085
Age (years)		−0.969	<0.001	−1.182	−0.755
Weight (kg)		−0.286	0.009	−0.502	−0.070
Physical activity	active	3.582	0.316	−3.424	10.589
Food (Kcal)		1.005	<0.001	0.997	1.012
Total beverages (100 g)		−0.889	<0.008	−1.542	−0.235
5caloricbeverages (100 g) *		40.079	<0.001	38.845	41.312

* “5caloricbeverages” = sum of hot beverages, milk, fruit & vegetable juice, caloric soft drinks and alcoholic drinks.

**Table A2:** Within-person change Model: estimated change in energy intake associated with beverage substitution.

**Model 3: Estimated Effect of Substituting Each Beverage Type ***				
**Changes in:**	**B**	***p* Value**	**95% Confidence Interval for B**	
			**Lower**	**Upper**
Hot beverages (100 g)	−29.080	0.016	−52.833	−5.326
Milk (100 g)	6.539	0.506	−12.726	25.804
Fruit & vegetable juice (100 g)	47.147	0.030	4.578	89.716
Caloric soft drink (100 g)	17.681	0.155	−6.706	42.069
Diet soft drink (100 g)	−35.621	0.069	−73.997	2.755
Alcoholic drinks (100 g)	18.612	0.027	2.128	35.096
Water (100 g)	−34.139	<0.0001	−42.263	−26.014
**Model 4: Estimated Effect of Caloric Beverages Replacing Non-Caloric Beverages**				
	**B**	***p* Value**	**95% Confidence Interval for B**	
			**Lower**	**Upper**
Change in total beverages (100 g)	−6.083	0.007	−10.485	−1.682
Change in caloric beverages (100 g)	43.387	<0.0001	34.702	52.071
**Model 5: Estimated Effect of Caloric Beverages Replacing Caloric Beverages, Holding Food Energy Constant**				
	**B**	***p* Value**	**95% Confidence Interval for B**	
			**Lower**	**Upper**
Change in food energy (100 kcal)	103.626	<0.0001	102.587	104.666
Change in total beverages (100 g)	−7.718	<0.0001	−8.701	−6.735
Change in caloric beverages (100 g)	34.232	<0.0001	32.292	36.173

* Model 6 is a composite of 7 regressions, one for each beverage, adjusted for change in total beverages.
